# P-2122. Assessment of Pre-operative Antibiotic Prophylaxis and Urine Culture Utilization in Invasive Urological Procedures: A Retrospective Review

**DOI:** 10.1093/ofid/ofaf695.2286

**Published:** 2026-01-11

**Authors:** Anila Hussain, Raquel Nahra, Alexandra Hanretty, Geena Kludjian, Alia McConnell, Dibato John Epoh

**Affiliations:** Rhode Island Hospital, East Greenwich, RI; Cooper University Health, Camden, New Jersey; Cooper University Hospital and Cooper Medical School of Rowan University, Philadelphia, Pennsylvania; Cooper University Hospital and Cooper Medical School of Rowan University, Philadelphia, Pennsylvania; Cooper University Health Care, Camden, New Jersey; Cooper University Health Care, Camden, New Jersey

## Abstract

**Background:**

Invasive procedures of the urogenital tract are associated with increased postoperative infection risk, particularly in those with a history of bacteriuria. The American Urological Association recommends a single dose of peri-procedural antibiotic prophylaxis for specific procedures. The objective of our study was to identify risk factors for post-operative complications to develop a standardized workflow for timely urine culture collection, review, and antibiotic selection

**Table 1.**
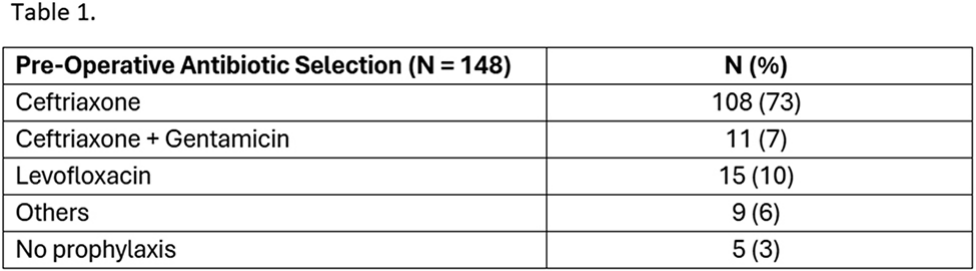
Pre-operative Antibiotic Selection

**Table 2.**
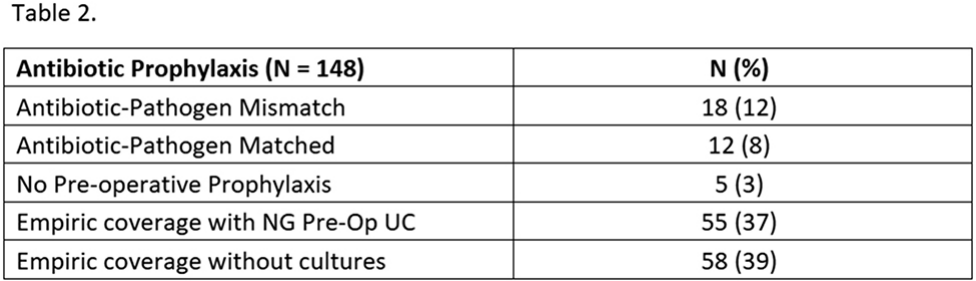
Antibiotic prophylaxis and Match rates

**Methods:**

We conducted a retrospective chart review of patients who underwent cystoscopy/cystourethroscopy with stent placement and/or lithotripsy between June and October 2023. Data collected included urinalysis (UA) and urine culture (UC), time from UC to procedure, antibiotic selection, and relevant microbiology data. Postoperative outcomes, including complications and readmissions, were assessed. Comparative analyses were performed to assess differences in clinical variables between patients with and without UC

**Table 3.**
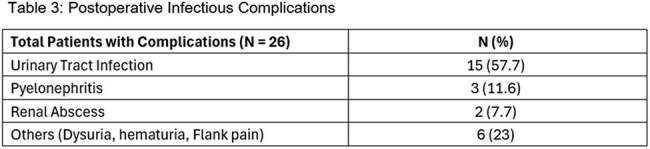
Post-operative Infectious Complications

**Results:**

A total of 148 patients were included in the analysis. Pre-operative UA was performed in 117 patients (79%), and a pre-operative UC was available within 60 days in 89 patients (60%). UA and/or UC were obtained about 2 to 3 weeks before surgery (median 9 days), with no significant difference in complications. Antibiotics used for prophylaxis were variable (Table 1). Antibiotic-pathogen mismatches were identified in 18 patients (12%) (Table 2). Postoperative infectious complications were noted in 24 patients (16%) (Table 3). Among 24 patients with complications, 5 had documented antibiotic mismatches, 10 lacked pre-operative culture data, 9 had negative urine cultures, and 2 had positive urine cultures with appropriate antibiotic coverage. Infection-related readmissions occurred in 17 patients (11%). All patients who were readmitted had experienced a postoperative complication (100% vs 7% in non-readmitted; p < 0.0001)

**Conclusion:**

This study identified key risk factors for post-operative complications, including a lack of pre-operative urine cultures and mismatched antibiotic coverage. A workflow to standardize urine culture protocols and ensure appropriate prophylaxis may help reduce preventable infections and enhance surgical outcomes.

**Disclosures:**

Alexandra Hanretty, PharmD, Abbvie: Honoraria

